# Infliximab-Associated Xanthogranulomatous Pyelonephritis: A Rare Complication

**DOI:** 10.7759/cureus.21051

**Published:** 2022-01-09

**Authors:** Adnan Alzanbagi, Ghadeer A Alhazmi, Shuruq Alghamdi, Ghaida Alosaimi, Mohammed K Shariff

**Affiliations:** 1 Gastroenterology, King Abdullah Medical City, Makkah, SAU; 2 Medicine, Umm Al-Qura University, Makkah, SAU

**Keywords:** xanthogranulomatous pyelonephritis, anti-tumor necrosis factor blocker, infliximab, ulcerative colitis (uc)

## Abstract

Xanthogranulomatous pyelonephritis (XGP) is one of the rare diseases characterized by chronic inflammation and destruction of the renal parenchyma, and it is usually associated with renal infection, and nephrolithiasis. Infliximab is an anti-tumor necrosis factor-alpha (anti-TNF-α) monoclonal antibody, which is widely used for treating inflammatory bowel disease, and it is known to increase the risk of rare and opportunistic infections. In this study, we report a case of XGP presenting after the initiation of infliximab treatment. We highlight one of the possible complications associated with immunosuppression due to infliximab. Furthermore, the importance of increasing the awareness among physicians for early recognition of this rare complication.

## Introduction

Xanthogranulomatous pyelonephritis (XGP) is one of the rare diseases characterized by chronic inflammation and destruction of the renal parenchyma structure caused by lipid-laden macrophages also known as xanthoma cells infiltration [1]. It commonly affects middle-aged women from 40 to 69 years [2]. Three different types of XGP are described as the majority are diffuse (90%), with segmental and focal together constituting around 13% of cases [3]. It is one of the challenging diseases to diagnose because the presentation is non-specific with variable clinical features, laboratory abnormalities, and radiological findings [4]. Infliximab is a genetically engineered monoclonal antibody targeted to inhibit the proinflammatory cytokine, tumor necrosis factor-alpha (TNF-α) [5]. It is one of the first-line biologics used for the treatment of a variety of inflammatory disorders like rheumatoid arthritis, psoriasis, Inflammatory bowel disease (IBD), ankylosing spondylitis, and it is widely used [5]. Though it is generally considered safe, however, it does increase the risk of serious infections, including opportunistic and rare infections. In addition, it is linked with an increased risk of malignancy, like lymphoma, and skin cancer. We present a rare case of xanthogranulomatous pyelonephritis (XGP) associated with infliximab therapy and highlight the importance for healthcare workers to think about the possibility of this association in patients on infliximab with unexplained fever.

## Case presentation

A 25-year-old Saudi woman, known case of ulcerative colitis (UC) diagnosed in 2015, was maintained on azathioprine. One year after the diagnosis due to loss of response to azathioprine monotherapy, infliximab was added as a combination every 8 weeks. However, a year after that she was admitted to our hospital, King Abdullah Medical City (KAMC) in Makkah with fever of 10 days duration. The fever continued and was associated with chills, rigors, and non-projectile vomiting. She had three bowel motions of soft brown stools, not associated with mucous or blood. She denied any symptoms of abdominal pain, dysuria, hematuria, frequency, or urgency. The systemic review was unremarkable. Past medical history included a horseshoe-shaped kidney that was discovered incidentally on a routine abdominal CT scan done two years ago. Apart from the above-mentioned medications, she was on no other drugs. There wasn’t any family history of malignancy or tuberculosis (TB). She was married and did not have any contact with TB patients. She had not traveled recently, and she never smoked.

The patient appeared ill with a temperature of 39°C, heart rate of 143 beats/minute, blood pressure of 107/52 mmHg, respiratory rate of 18/minute, and oxygen saturation of 100% on air. She looked dehydrated, pale, with no peripheral lymphadenopathy. Her systemic examination was unremarkable. The patient’s laboratory data on admission are shown in Table 1. Urine examination showed traces of protein and blood, red blood cells 5-10/high power field, white cell count 0-2/high power field, no red blood cell casts or dysmorphic cells. Nitrite and leukocyte esterase were negative. Stool examination, blood culture, urine culture, Epstein-Bar virus, cytomegalovirus (CMV), brucella serology, and acid-fast bacilli on three early morning sputum samples were negative (Table 1). The chest x-ray of the patient was normal.

**Table 1:** Patients’ liver function test, stool examination, inflammatory marker, complete blood count (CBC), renal function test and, other workups. TB: tuberculosis; CMV: cytomegalovirus; HBsAg: hepatitis B surface antigen; HCV: hepatitis C virus antibody; HIV: human immunodeficiency virus; EBV: Epstein-Bar virus; CRP: C-reactive protein; ESR: erythrocyte sedimentation rate; PCR: polymerase chain reaction

Laboratory data		
Liver function test	Total bilirubin	6.48 mg/dL
	Direct bilirubin	0.37 mg/dL
	Aspartate transaminase (AST)	38 U/L
	Alanine transaminase (ALT)	9 IU
	Alkaline phosphatases	79 U/L
	Albumin	2.85 g/dL
	Gamma-glutamyltransferase	14 U/L
	International normalized ratio(INR)	0.99
Renal function test	Serum creatinine	2.9 mg/dl
	Blood urea nitrogen	37 mg/dl
Inflammatory marker	ESR	80 mm/h
	CRP	35.4 mg/dl
CBC	Hemoglobin	11.9 g/dl
	White blood cell (WBC)	9480/mm^3^
	Platelet count	315,000/mm
Stool examination	Color and consistency	Soft brown
	RBC	Negative
	Blood	Negative
	WBC	Negative
	Pus	Negative
	Epithelial cells	Negative
	Mucus	Negative
	Helminths	Negative
Other workups	TB by PCR on renal tissue	Negative for *Mycobacterium tuberculosis*, *Mycobacterium africanum*, *Mycobacterium bovis*, *Mycobacterium microti*, *Mycobacterium pinnipedii*
	Malaria PCR	Negative
	CMV	Negative
	HBsAg	Negative
	HCV	Negative
	HIV screening antigen-antibody complex	Negative
	EBV	Negative
	Brucella	Negative
	Blood culture	Negative
	Urine culture	Negative

**Figure 1:**
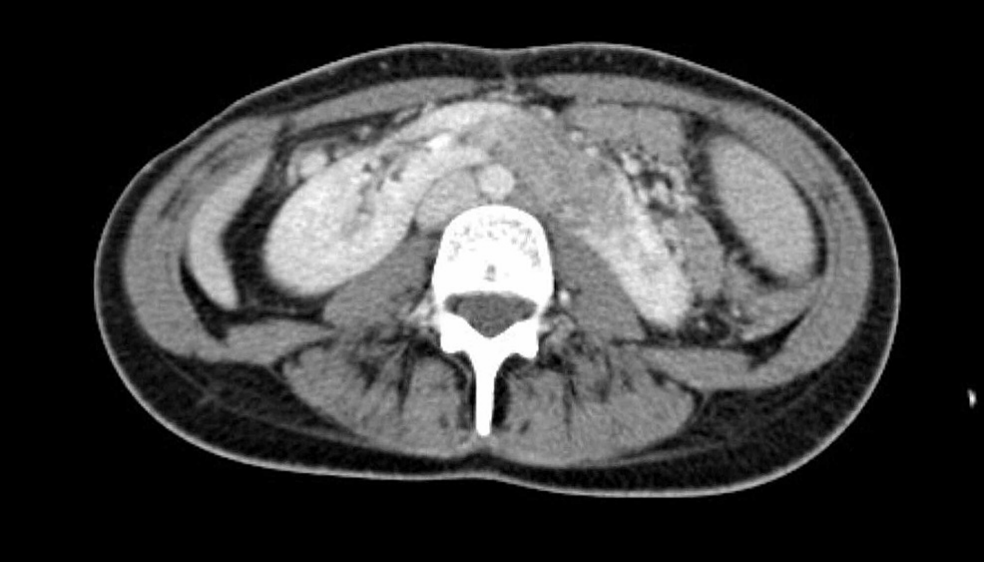
Abdominal CT scan without contrast shows horseshoe-shaped kidneys with suspicion of renal mass.

Colonoscopy revealed mild colitis that was confirmed on histopathology report. There were no granulomas, dysplasia, or CMV inclusion bodies seen on biopsy. CT scan for the abdomen showed horseshoe-shaped kidney in addition to heterogeneous hypodense soft tissue mass in the lower pole of left kidney crossing the midline measuring 5.5 x 2 x 4 cm, with multiple variable size abscesses involving the left lower kidney (Figure 1).

**Figure 2:**
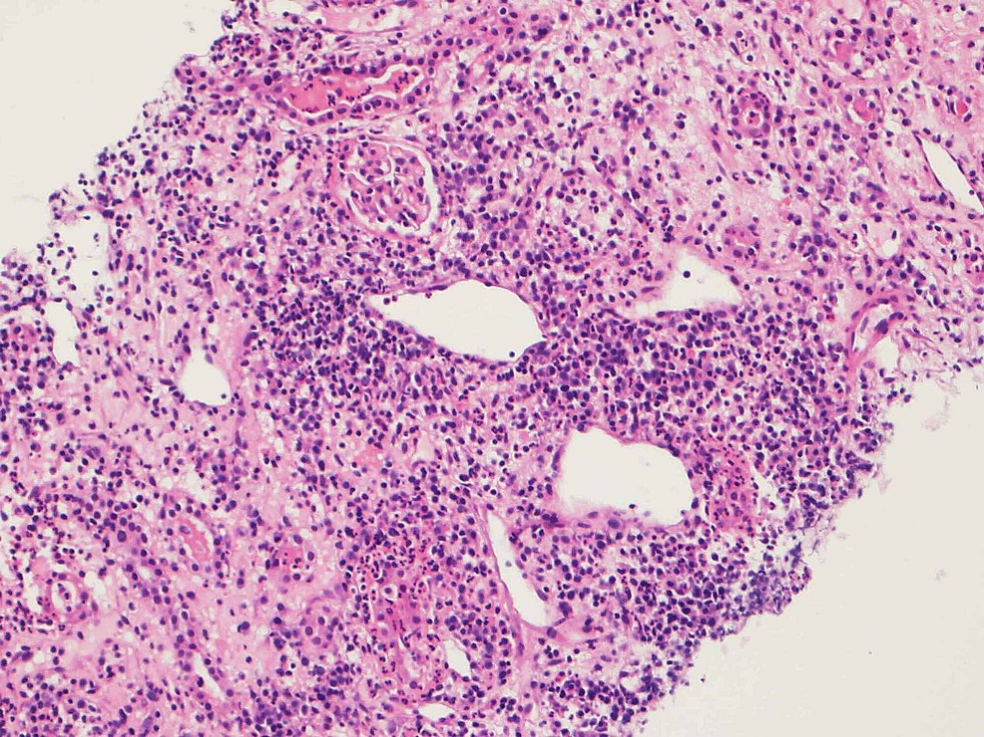
Renal biopsy revealed features of chronic inflammation of the kidney. The interstitium shows moderate infiltration of mixed inflammatory cells comprised of lymphocytes, plasma cells, many histiocytes, eosinophils, and some neutrophils with focal area of necrosis.

Due to the presence of a mass, a CT-guided renal mass biopsy was done, which showed linear cores of renal tissue with viable and intact glomeruli. Interstitium showed moderate infiltration of mixed inflammatory cells comprising of lymphocytes, plasma cells, mainly histiocytes, eosinophils, and some neutrophils. Focal area of necrosis was also present. In some areas, lymphocytes and neutrophils were seen invading the tubular epithelium, and neutrophilic cast was noted within the tubular lumen (Figure 2).

No evidence of granuloma, lymphoma, or malignancy was seen. Special stains of Ziehl Neelsen and periodic acid-Schiff diastase were done to rule out mycobacterial and fungal infection and were negative. In addition, a renal tissue polymerase chain (PCR) reaction and culture for tuberculosis (TB) were negative. After these investigations, the patient was diagnosed to have xanthogranulomatous pyelonephritis.

Since infliximab is known to cause reactive TB, the diagnosis of TB was ruled out in our case. A normal chest x-ray, absence of acid-fast bacilli in sputum, with a negative PCR and culture for TB on renal tissue excluded both systemic and renal TB. The possibility of fever due to ulcerative colitis per se and opportunistic infections like CMV were considered. However, with a picture of mild colitis on colonoscopy and the absence of inclusion bodies, these were excluded. Due to the potential association of infliximab with lymphoma, this differential was considered. CT scan did not show any significant lymphadenopathy to raise the suspicion of lymphoma. However, the finding of renal mass raised the possibility of renal cancer, this prompted the renal biopsy that confirmed the diagnosis of XGP.

**Figure 3:**
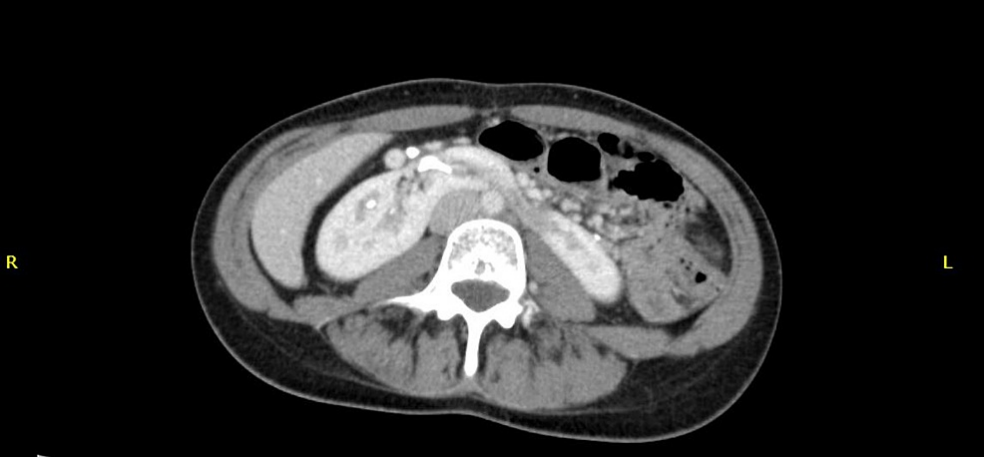
Abdominal CT scan without contrast shows resolution of abscesses.

Infliximab was stopped, and azathioprine was continued as maintenance therapy for UC. As no source of infection was found, the patient was initially treated with ceftriaxone 1 g empirically for a week. Following the CT finding of mass and abscess with continued pyrexia, the antibiotic was changed to meropenem. It was empirically decided to continue this antibiotic for five weeks, and assess the response. During these five weeks, the temperature gradually normalized, and a CT abdomen at the end of five weeks showed regression of the renal mass with the resolution of abscesses (Figure 3). She was followed up in the outpatient clinic a month later. There was no more fever reported, and clinical examination was unremarkable with a normal temperature.

## Discussion

XGP is a rare disease with a high incidence among females with non-specific manifestation, and varied presentation, making it difficult to diagnose [6]. The etiology and pathogenesis of XGP are not clear and usually present on a background of urinary infection, obstruction, and renal stones [7]. Histopathology is the cornerstone for the diagnosis of XGP as it mimics other pathological diseases, with only around 20% of cases being diagnosed preoperatively [6,7]. One series of XGP reported congenital pelvic ureteric junction anomaly as a predisposing factor in 11% of its cases [8]. Most XGP are treated surgically either due to misdiagnosis or as definite therapy, antibiotics are rarely effective [6-8]. Infliximab is an anti-tumor necrosis factor biologic, that is recommended for the treatment of moderate to severe ulcerative colitis, and it is commonly prescribed [9]. Due to its immunosuppressive action, it increases the risk of serious infections like reactivation of tuberculosis and invasive opportunistic infections [10]. In addition, it increases the risk of non-Hodgkin's lymphoma and auto-immune diseases [11]. These risks are further increased when it is used in combination with azathioprine [10,11]. However, no one has reported a case of XGP in association with infliximab before. The temporal relationship with infliximab and no radiological evidence of XGP on previous CT makes it more likely that XGP is associated with infliximab. Furthermore, radiological improvement following the withdrawal of infliximab supports this association. Though antibiotics may have been the cause of amelioration, however, it has to be borne in mind that rarely are antibiotics effective in treating XGP, as noted above. It is plausible that preexisting congenital anomaly and immunosuppression from infliximab therapy may have predisposed the development of XGP. Association with azathioprine is less likely because XGP improved despite the continuation of azathioprine. To ascertain whether XGP was an infliximab-related adverse event, we used the WHO-Uppsala monitoring center case causality assessment tool for suspected drug adverse reaction [12]. Based on the causality categories, XGP was "probably" related to infliximab (Table 2).

**Table 2:** WHO-Uppsala monitoring center causality categories.

Causality term	Assessment criteria
Certain	Event happened at the same duration of drug intake; unexplainable by any other drugs or disease; response to withdrawal plausible (pathologically, pharmacologically); event definitive phenomenologically or pharmacologically; rechallenge is satisfactory
Probable/likely	Event happened at the same duration of drug intake; unlikely to be caused to any drugs or disease; response to withdrawal clinically reasonable; not required rechallenge
Possible	Event happened at the same duration of drug intake; explained by other disease or drug; lacking and unclear information on drug withdrawal
Unlikely	The relationship when the event at the same time to drug intake is improbable (but not impossible); drugs or diseases other provide plausible explanations
Conditional/unclassified	Abnormal event; additional data are needed for better assessment; more data under examination
Unassessable/unclassifiable	Adverse reactions are reported; could not be judged because of insufficient data; verified data

## Conclusions

Our case highlights the association of XGP with infliximab, and this should be considered in patients who have an unexplained fever with urinary symptoms. Apart from infliximab, other anti-TNF biologics may potentially increase the risk of XGP due to similar mode of action. Hence, the prescription of these drugs in patients with predisposing factors for XGP like renal stones and renal anomalies should be carefully weighed against the benefit.

## References

[1] Hayashi Y, Kawahara T, Hattori Y (2017). Xanthogranulomatous pyelonephritis with incomplete double ureter. Case Rep Med.

[2] Butticè S, Antonino I, Giorgio A, Valeria B, Stefano P, Giuseppe M, Carlo M (2014). Xanthogranulomatous pyelonephritis can simulate a complex cyst: case description and review of literature. Urol Case Rep.

[3] Loffroy R, Guiu B, Watfa J, Michel F, Cercueil JP, Krausé D (2007). Xanthogranulomatous pyelonephritis in adults: clinical and radiological findings in diffuse and focal forms. Clin Radiol.

[4] Mirza MU, Van Taunay J, Waleed M, Vangimalla SS, Hegde S, Moustafa MA (2019). Xanthogranulomatous pyelonephritis: synchronous upper and lower gastrointestinal bleed. J Investig Med High Impact Case Rep.

[5] Travassos WJ, Cheifetz AS (2005). Infliximab: use in inflammatory bowel disease. Curr Treat Options Gastroenterol.

[6] Korkes F, Favoretto RL, Bróglio M, Silva CA, Castro MG, Perez MD (2008). Xanthogranulomatous pyelonephritis: clinical experience with 41 cases. Urology.

[7] Kim SW, Yoon BI, Ha US, Sohn DW, Cho YH (2013). Xanthogranulomatous pyelonephritis: clinical experience with 21 cases. J Infect Chemother.

[8] Addison B, Zargar H, Lilic N, Merrilees D, Rice M (2015). Analysis of 35 cases of xanthogranulomatous pyelonephritis. ANZ J Surg.

[9] Rubin DT, Ananthakrishnan AN, Siegel CA, Sauer BG, Long MD (2019). ACG clinical guideline: ulcerative colitis in adults. Am J Gastroenterol.

[10] Van Assche G, Lewis JD, Lichtenstein GR (2011). The London position statement of the World Congress of Gastroenterology on Biological Therapy for IBD with the European Crohn's and Colitis Organisation: safety. Am J Gastroenterol.

[11] Shivaji UN, Sharratt CL, Thomas T (2019). Review article: managing the adverse events caused by anti-TNF therapy in inflammatory bowel disease. Aliment Pharmacol Ther.

[12] (2018). The use of the WHO-UMC system for standardised case causality assessment. https://www.who-umc.org/media/164200/who-umc-causality-assessment_new-logo.pdf.

